# Generating missing patient anatomy from partially acquired cone-beam computed tomography images using deep learning: a proof of concept

**DOI:** 10.1007/s13246-023-01302-y

**Published:** 2023-07-18

**Authors:** Benjamin Shields, Prabhakar Ramachandran

**Affiliations:** 1 Biomedical Technology Services, Townsville University Hospital, Townsville, Australia; 2 School of Chemistry and Physics, Queensland University of Technology, Brisbane, Australia; 3 Department of Radiation Oncology, Princess Alexandra Hospital, Brisbane, Australia

**Keywords:** Cone-beam computed tomography, Machine learning, Radiotherapy

## Abstract

The patient setup technique currently in practice in most radiotherapy departments utilises on-couch cone-beam computed tomography (CBCT) imaging. Patients are positioned on the treatment couch using visual markers, followed by fine adjustments to the treatment couch position depending on the shift observed between the computed tomography (CT) image acquired for treatment planning and the CBCT image acquired immediately before commencing treatment. The field of view of CBCT images is limited to the size of the kV imager which leads to the acquisition of partial CBCT scans for lateralised tumors. The cone-beam geometry results in high amounts of streaking artifacts and in conjunction with limited anatomical information reduces the registration accuracy between planning CT and the CBCT image. This study proposes a methodology that can improve radiotherapy patient setup CBCT images by removing streaking artifacts and generating the missing patient anatomy with patient-specific precision. This research was split into two separate studies. In Study A, synthetic CBCT (sCBCT) data was created and used to train two machine learning models, one for removing streaking artifacts and the other for generating the missing patient anatomy. In Study B, planning CT and on-couch CBCT data from several patients was used to train a base model, from which a transfer of learning was performed using imagery from a single patient, producing a patient-specific model. The models developed for Study A performed well at removing streaking artifacts and generating the missing anatomy. The outputs yielded in Study B show that the model understands the individual patient and can generate the missing anatomy from partial CBCT datasets. The outputs generated demonstrate that there is utility in the proposed methodology which could improve the patient setup and ultimately lead to improving overall treatment quality.

## Introduction

In conventional radiotherapy, cancer patients often receive a fractionated dose of radiation, with the aim of minimising toxicity and improving the effectiveness of tumor cell eradication. This is supported by the principles of radiobiology which include the 5Rs; repair, repopulation, reoxygenation, redistribution, and intrinsic radiosensitivity [1–3]. In a typical fractionation schedule, the radiation dose is divided into 20 to 30 daily treatment sessions, with the cumulative dose in each fraction totalling the prescribed dose. While this approach leads to manageable toxicity, it introduces new challenges such as the potential for patient setup error due to the need for multiple fractions [4–6]. Radiotherapy linacs are designed to deliver precise amounts of radiation to the same volume within the body with high reproducibility for each fraction [7]. Therefore, it is essential that the patient is set up in the exact same location and orientation for each fraction. Previously, patients were positioned using lasers fixed in the walls of the treatment room which were aligned with crosshairs tattooed on the patient’s body [8, 9]. While the tattoos remain fixed in place on the patient’s skin, the internal anatomy is constantly moving with the patient’s physiology i.e., respiration, organ function, and fullness of the stomach/bladder/bowel [10, 11].

**Fig. 1:**
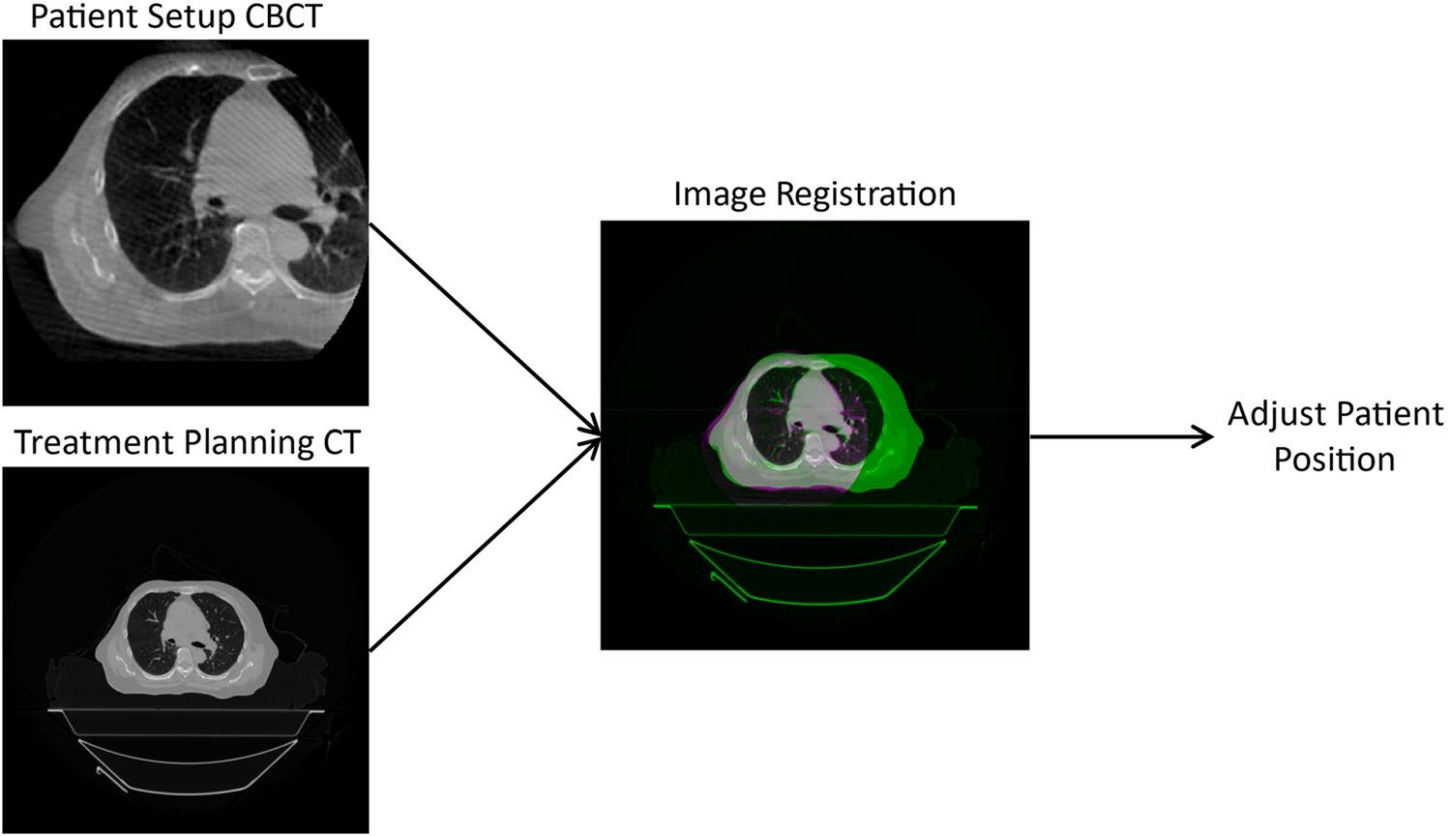
Current industry standard for setting up a radiotherapy patient in the correct position for treatment

In modern radiotherapy treatment machines, a kilo-voltage x-ray beam is used to obtain a CBCT image of the patient while they are on the treatment couch [12]. This image is then registered to the planning CT images using software that matches the patient’s anatomy in both images [13, 14]. The registered CBCT images are then used to ensure that the patient is in the correct position and orientation for treatment. This is achieved by comparing the registered images with the planning images and making any necessary adjustments to the patient’s position, such as repositioning the treatment couch or moving the patient’s body [7]. Once the patient is properly aligned, the radiation treatment can be delivered with high precision and accuracy. At present, all modern radiotherapy machines using the CBCT technique for patient setup and are doing so using partially acquired images. Figure 1 shows a typical CBCT to CT registration where the entire patient anatomy does not fit within the field of view of the CBCT scanner.

The quality of the CBCT images has become essential to the accuracy of patient setup, thus research into improving CBCT images is increasingly common [15]. CBCT images have two main shortcomings which limit their planning CT registration accuracy. Firstly, due to the cone-beam geometry and the presence of high-density materials such as metallic implants or dental fillings, CBCT images suffer from an increased amount of x-ray scatter which results in high amounts of streaking artifacts [16, 17]. These artifacts can lead to inaccurate dose calculations and impair the ability to accurately target the tumor, which can compromise the success of the radiotherapy treatment. Secondly, CBCT images have a field of view that is not typically large enough to include the entire patient’s cross-section, leading to part of the patient’s anatomy not being captured particularly if the tumour is lateralised [18–20].

Artificial intelligence (AI) techniques have been developed to reduce streaking artifacts on CBCT images, and several studies have reported promising results. One approach is to use deep learning algorithms, such as convolutional neural networks (CNNs), to learn the underlying patterns of the artifacts and remove them from the images. Several studies have shown that a CNN-based method can effectively reduce streaking artefacts in CBCT images while preserving image details [21, 22]. Another approach is to use iterative reconstruction algorithms, such as model-based iterative reconstruction (MBIR) to reduce artifacts [23–25].

**Fig. 2:**
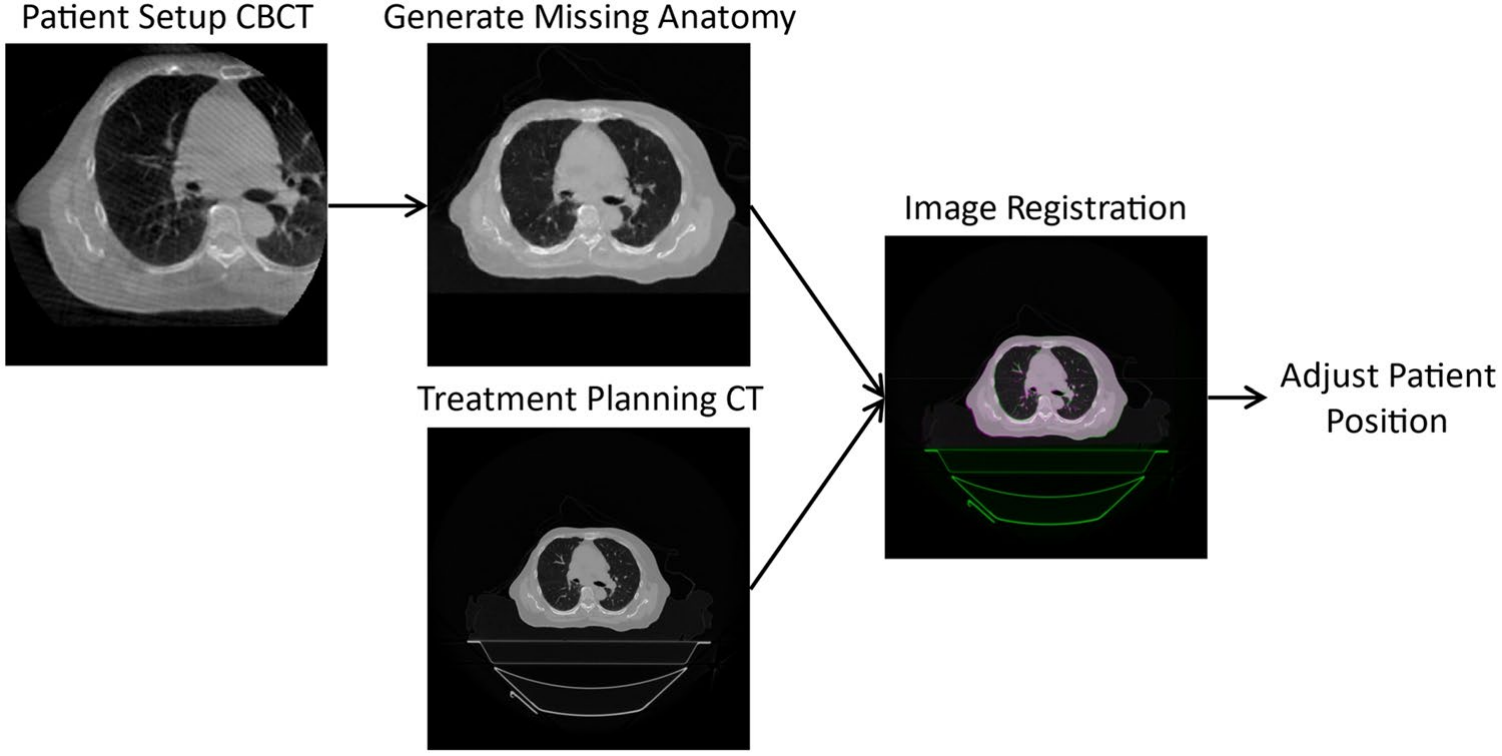
The proposed workflow. Incorporation of a machine learning model which generates the anatomy not captured by typical on-couch CBCT imaging to aid image registration to the planning CT

This study explores the use of a machine learning algorithm to firstly, remove image artifacts and secondly, to increase the field of view of CBCT images and generate the full patient anatomy. The aim of this research was to use the output image to set up a patient more accurately for a radiotherapy fraction than what the regular CBCT is capable of providing. Figure 2 demonstrates the proposed workflow.

This research was completed as two sequential studies. Study A used sCBCT images to train two machine learning models: one model for artefact removal, and the other to generate the missing patient anatomy (image imputation). Study B used patient CBCT and planning CT images to train a single machine-learning model to remove artifacts and generate patient anatomy outside the field of view of the CBCT image.

## Materials and methods

### Study A

Training image datasets for Study A included publicly available, anonymised CT images collected by United States researchers for the National Lung Screening Trial. The trial enrolled 53,454 people and compared the effectiveness of detecting lung cancer using a chest X-ray compared to a low-dose CT scan [26]. 422 of the CT volumes captured for this trial are available online [27]. To decrease variability in training data, the dataset was reduced by only utilising images of the same dimensions, taken on the same model of CT scanner including images of the thoracic region only. All images are 512 × 512 and were taken on a Siemens Biograph 40 PET CT Scanner, with CT volumes cropped to exclude anatomy above and below the thoracic region. This significantly reduced the size of the dataset while simultaneously increasing its quality. After completing pre-processing of the data, the dataset was reduced to a total of 6200 CT images from 168 patients. 6100 images for training and a further 100 for testing.

Machine learning models were developed using the pix2pix Conditional Generative Adversarial Network (CGAN) implemented in MATLAB [28, 29]. The advantage of this network is translational ability. This allows the algorithm to receive an input image containing limited information and based on the image’s underlying features, generates a plausible output. Description of the network layers can be found in Table 1.

**Table 1:** Description of individual layers within the pix2pix generator model

Layer #	Layer description	Layer #	Layer description	Layer #	Layer description
1	Image Input Layer	21	Leaky ReLU Layer	41	Batch Normalization Layer
2	Convolution 2D Layer	22	Convolution 2D Layer	42	Dropout Layer
3	Leaky ReLU Layer	23	Batch Normalization Layer	43	Leaky ReLU Layer
4	Convolution 2D Layer	24	Leaky ReLU Layer	44	Depth Concatenation Layer
5	Batch Normalization Layer	25	Transposed Convolution 2D Layer	45	Transposed Convolution 2D Layer
6	Leaky ReLU Layer	26	Batch Normalization Layer	46	Batch Normalization Layer
7	Convolution 2D Layer	27	Dropout Layer	47	Leaky ReLU Layer
8	Batch Normalization Layer	28	Leaky ReLU Layer	48	Depth Concatenation Layer
9	Leaky ReLU Layer	29	Depth Concatenation Layer	49	Transposed Convolution 2D Layer
10	Convolution 2D Layer	30	Transposed Convolution 2D Layer	50	Batch Normalization Layer
11	Batch Normalization Layer	31	Batch Normalization Layer	51	Leaky ReLU Layer
12	Leaky ReLU Layer	32	Dropout Layer	52	Depth Concatenation Layer
13	Convolution 2D Layer	33	Leaky ReLU Layer	53	Transposed Convolution 2D Layer
14	Batch Normalization Layer	34	Depth Concatenation Layer	54	Batch Normalization Layer
15	Leaky ReLU Layer	35	Transposed Convolution 2D Layer	55	Leaky ReLU Layer
16	Convolution 2D Layer	36	Batch Normalization Layer	56	Depth Concatenation Layer
17	Batch Normalization Layer	37	Dropout Layer	57	Transposed Convolution 2D Layer
18	Leaky ReLU Layer	38	Leaky ReLU Layer	58	Batch Normalization Layer
19	Convolution 2D Layer	39	Depth Concatenation Layer	59	Leaky ReLU Layer
20	Batch Normalization Layer	40	Transposed Convolution 2D Layer	60	Depth Concatenation Layer
				61	Convolution 2D Layer

#### Creation of synthetic CBCT images

**Fig. 3:**
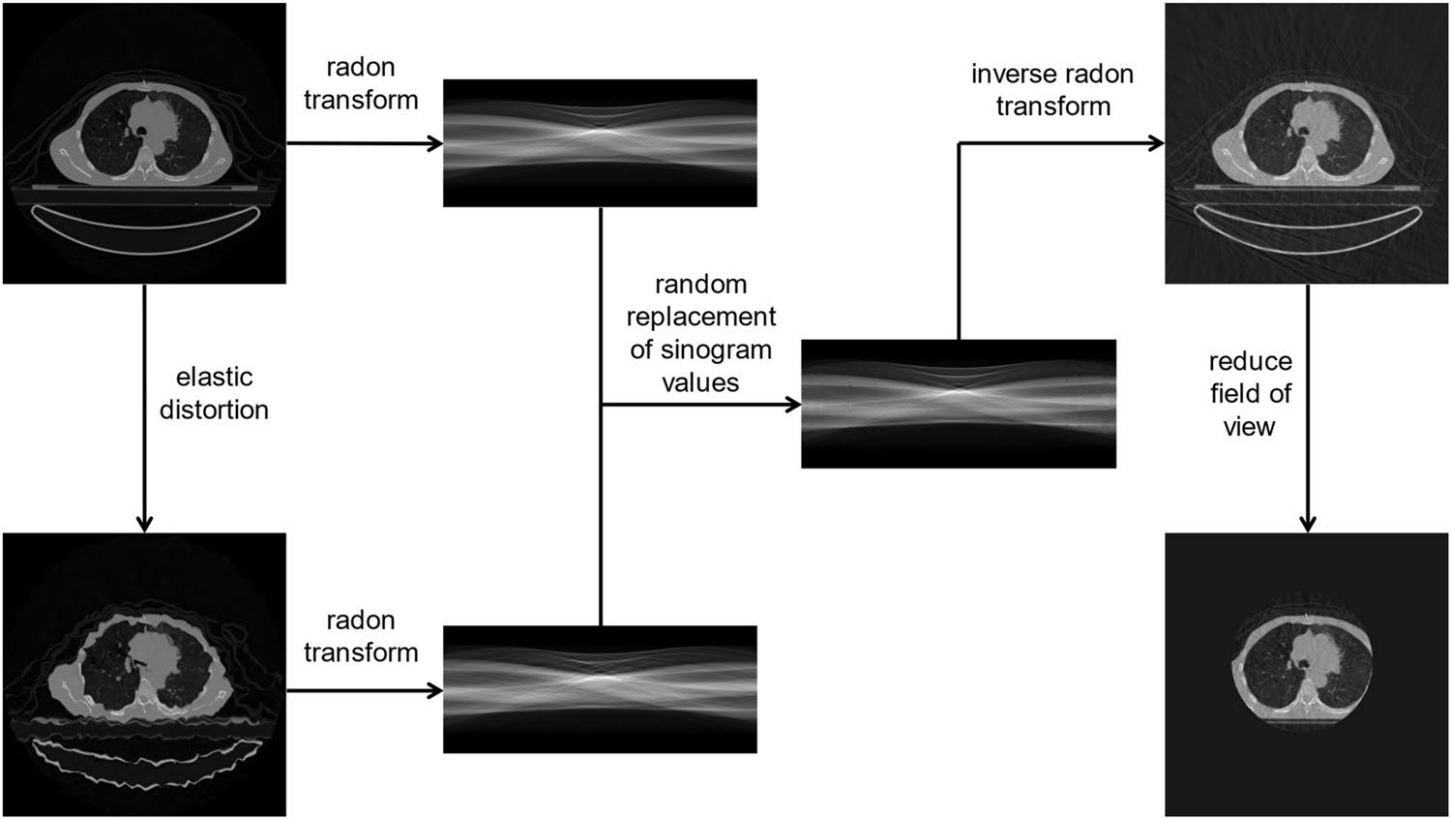
Creation of synthetic CBCT images

CBCT images were simulated by adding streaking artifacts to the ground truth CT image and then reducing the field of view. Firstly, a random elastic deformation matrix was applied to the image to create distortion. A radon transform was then performed on both the original image and the distorted image to produce the image sinograms. Using a random replacement of sinogram values, up to 10% of the original image sinogram was replaced with the distorted image sinogram. An inverse radon transform was applied to the combined image sinogram to produce original CT images with additional streaking artifacts [21]. The field of view was reduced by randomly selecting a isocentre which would result in partial CBCT acquisition should the subject be set up on a radiotherapy treatment couch. Pixels located beyond 128.px from the isocentre were assigned a value of zero to simulate a partial acquisition. The images were not recentered and padding was not reduced to simulate the sCBCT already being registered to the original CT image. A diagram showing the creation of the sCBCT images can be seen in Fig. 3.

#### Training data: streak-removal model

During the creation of the sCBCT images, the original CT image was cropped with the same dimensions to form the ground truth dataset to train the streak-removal model. Training was executed for 200 epochs (~ 10 days), after which, the outputs yielded were almost indistinguishable from the ground truth. At this point, the model was saved and was used to create the input dataset to train the image imputation model.

#### Training data: image imputation model

The output of the streak-removal model was used as the input to the image imputation model which was again trained for 200 epochs (~ 10 days). The image imputation model receives the output from the streak-removal model and based on the partial anatomy generates the missing anatomy. While the purpose of the image imputation model was not to remove artifacts, given that the input data was the output of the streak-removal module, and the output was the original CT, naturally, the model attempted to remove any remaining artifacts left behind by the streak-removal model as well as generate the missing data. Study A was performed in MATLAB 2022b on a Windows 10 machine with 8GB of RAM, i7 6700k CPU and a GTX 1060 6GB graphics card.

### Study B

After observing the streak-removal and image imputation models’ superior capability to accurately transform the sCBCT image into the original CT image, the next logical stage of this research was to develop a model using real patient image data. In the proof of concept using synthetic data, two models were produced, one to remove streaking artifacts and one for image imputation. Retaining this methodology for Study B would require cropping the planning CT to the exact dimensions of the registered CBCT to create the ground truth dataset. For this reason, the methodology for Study B was revised to eliminate the need for augmenting the ground truth dataset and a single model capable of removing artifacts and imputing the missing anatomy was trained.

A machine learning model which can generate missing image data will only be useful if it is accurate. Given that every patient has a unique anatomy or perhaps anatomical variations, how accurate can the model be? A one-size-fits-all approach to this problem is guaranteed to produce images of unsatisfactory accuracy for clinical use. The methodology used in Study A was revised to overcome this flaw. The methodology in Study B was to train a ‘base’ machine learning model using CT/partial CBCT image pairs from several patients and then perform a transfer of learning on a single patient’s image data to produce a patient-specific model. This new model with the application of transfer learning can receive partial CBCT input images and generate the missing anatomy specific to that patient.

Training data consisted of treatment planning CT and setup CBCT image sets showing partial anatomy for 15 patients who underwent treatment for non-small cell lung cancer (NSCLC). Three CBCT sets and one planning CT from a 16th patient was used for transfer learning and testing. All ground truth CT images were acquired on a radiotherapy CT scanner and input CBCT images were acquired on an Elekta XVI CBCT scanner. In total, the training dataset included 1365 image pairs and the transfer learning dataset included 98 image pairs and a further 49 image pairs for testing.

#### Pre-processing of clinical data

**Fig. 4:**
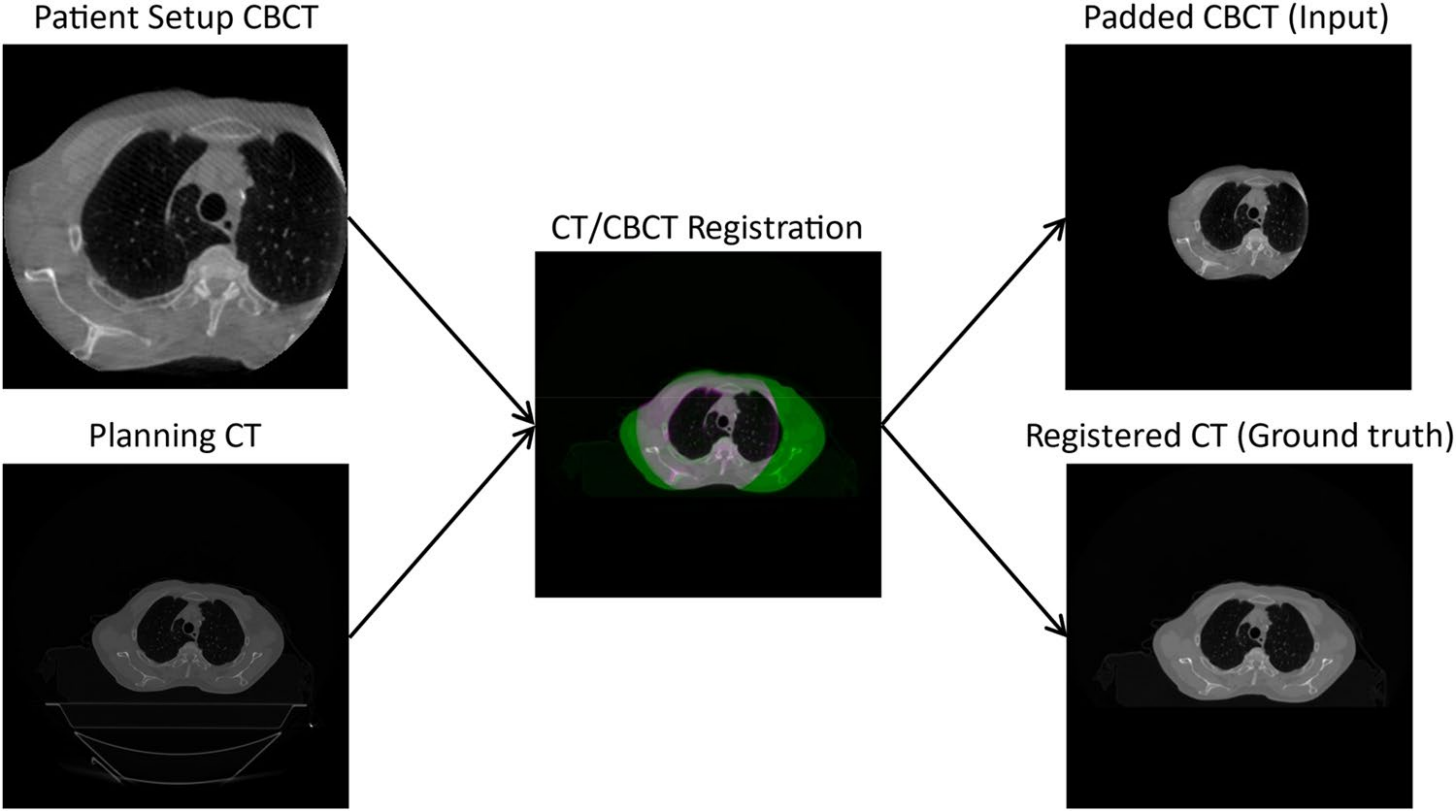
Creation of the padded CBCT and registered CT for input to the base clinical model

When collecting image volumes for the purpose of machine learning, it is essential that both the image sets have the same slice thickness to ensure that for each CBCT image, there is a corresponding ground truth CT image. For each image pair, the CBCT was padded with 0s to allow the registration of the CT image. The CT image was resized and then registered to the padded CBCT image. The couch was removed from the CT image by assigning a value of − 1024 to all pixels below the surface of the couch. Figure 4 shows a diagram of this workflow.

The base clinical model was trained for a total of 500 epochs (~ 2 days), after which a transfer of learning was performed on a single patient’s image data to produce a patient-specific model.

#### Producing patient-specific models via transfer learning

Transfer learning was achieved by taking the ‘base’ model and retraining the weights in only the final two layers of the machine learning network. Performing a transfer of learning is minimally computationally expensive for this reason as it is not producing an entirely new model, just a variation of the base model. The transfer of learning was initially completed using a single patient’s planning CT and one setup CBCT image set which included a total of 49 image pairs. Transfer learning was performed for 500 epochs (~ 3 hours) to create a variation of the model which is capable of producing a reasonable estimation of the missing anatomy as can be seen in Fig. 6. The model produced after transfer learning using one CBCT dataset is regarded as ‘patient-specific model 1’. Following this, transfer of learning was performed on the base clinical model again using the same patient’s planning CT data and two setup CBCT image sets which therefore doubled the transfer learning training data from 49 to 98 image pairs. This model was also trained for 500 epochs (~ 4 hours). This model is regarded as ‘patient-specific model 2’. Increasing the training data resulted in an improvement of the accuracy of the model’s output shown in Fig. 6. Each patient-specific model was tested on input CBCT images not previously seen by either model. Study B was performed in MATLAB 2023a on a Windows 11 PC running 16GB of RAM, i5 12400F CPU and a GTX 3060 12GB graphics card.

## Results

**Fig. 5:**
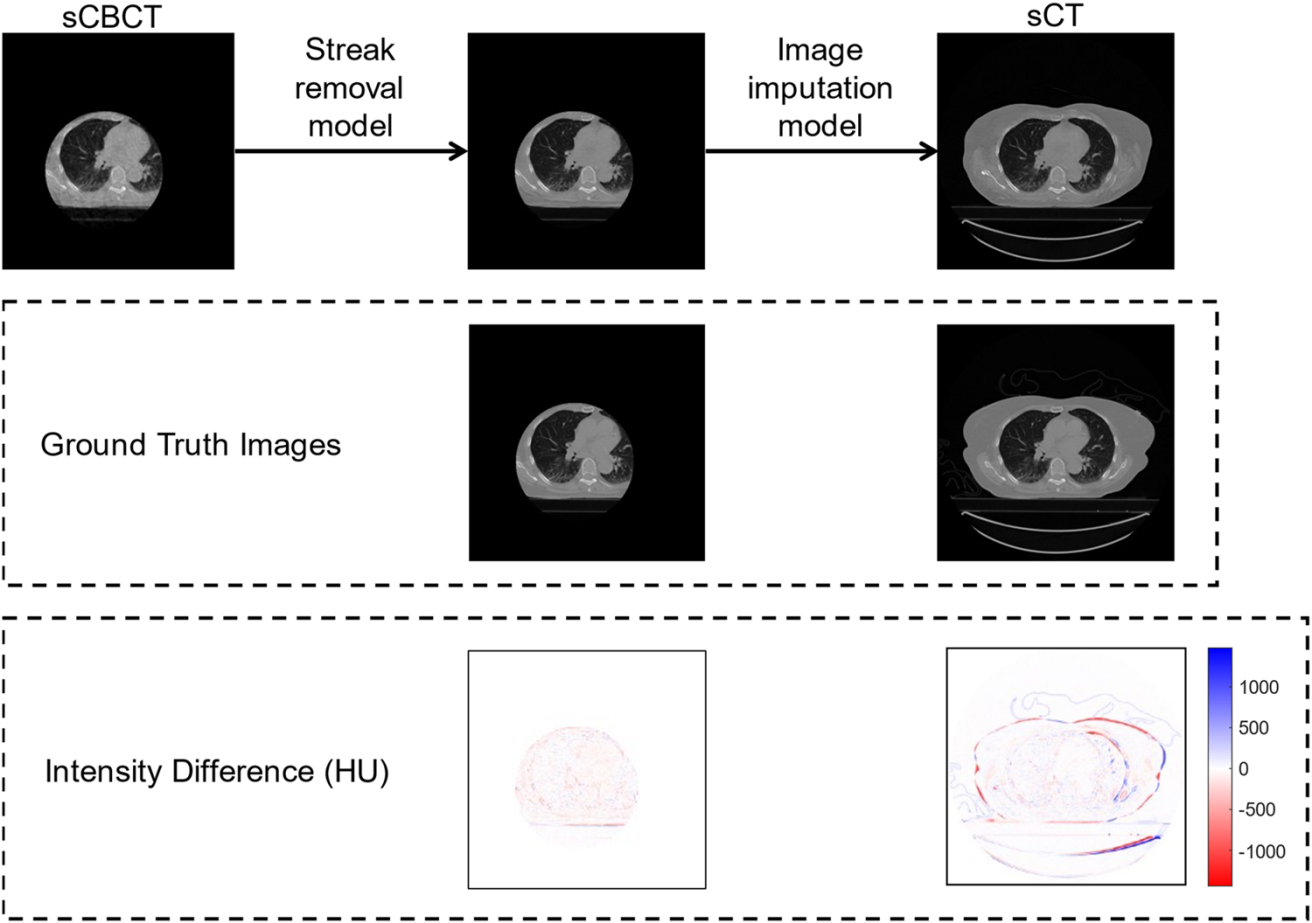
Performance of the streak-removal and image imputation models measured against the corresponding ground truth images. Intensity difference maps were creating by subtracting the sCT pixel values from the from the ground truth pixel values

Demonstrated in Fig. 5, the streak-removal model performs well at retaining most of the detail in the input image and the image imputation model does an impressive job at estimating the missing anatomy. The streak-removal model does appear to lose contrast between fat, soft tissue, and muscle. This is also true for the image imputation model in which there is a further loss of contrast in these areas. Aside from this loss of contrast, both models perform well at their respective tasks. It is evident that the model struggles particularly with generating bone and correctly estimating the edges of the patient and couch position. Regardless of these imperfections, the outputs generated by these models show utility in the methodology.

### Performance of patient-specific models

**Fig. 6:**
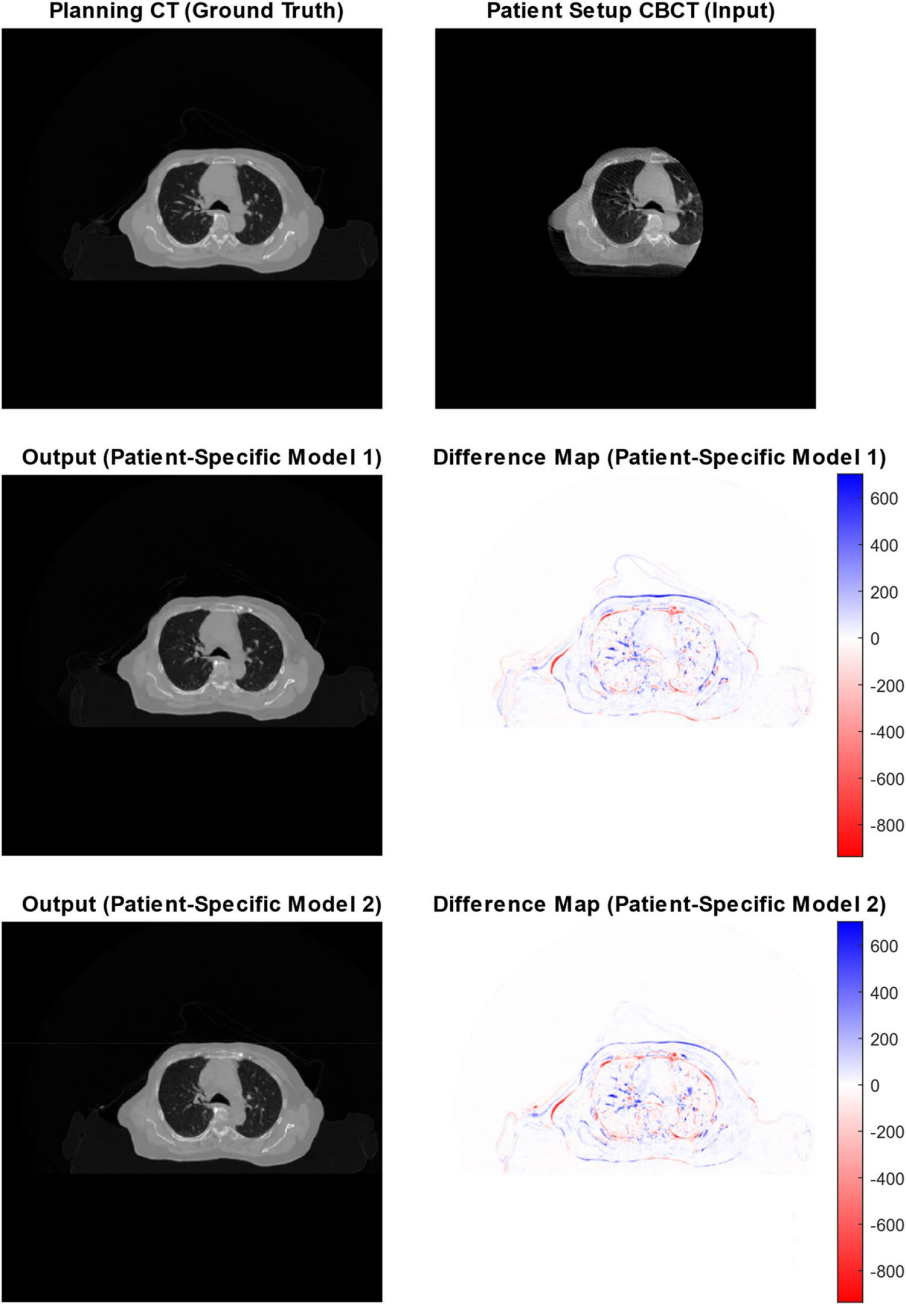
Performance of patient-specific models after 500 epochs of transfer learning. The difference maps were created by subtracting the model output image from the ground truth image. Colour scale is in Hounsfield Units (HU)

Figure 6 shows the performance of the patient-specific models. The input CBCT images used in Fig. 6 were kept the same in order to directly compare the performance of both models. As can be seen in the aforementioned figure, The patient-specific models are capable of producing anatomically plausible images with patient-specific precision and could perhaps rival the patient setup accuracy of the partial CBCT input image. The model can estimate the edges of the patient accurately but does appear to have difficulty in accurately generating the patient’s sternum. Structural Similarity Index Measure (SSIM) and Mean Absolute Error (MAE) were used to compare the accuracy of the patient-specific models vs. the regular CBCT and was calculated across the entire testing volume and averaged. Increasing the number of images pairs used for transfer learning resulted in a small increase in image quality, SSIM and MAE. The mean SSIM, MAE and respective standard deviations can be found in Table 2.

**Table 2:** Quantitative performance of the patient-specific machine learning models as compared with the regular CBCT currently used for patient setup

Image volume	SSIM	Standard deviation	MAE	Standard deviation
Regular CBCT	0.9993	$1.185\times {10}^{-4}$	72.8	1.79
Patient-specific model 1	0.9996	$6.931\times {10}^{-5}$	13.5	2.70
Patient-specific model 2	0.9997	$4.541\times {10}^{-5}$	12.2	2.42

## Discussion

Cone-beam computed tomography is a valuable tool in radiotherapy for patient setup and treatment planning. It provides high-resolution, 3D images of the patient’s anatomy, allowing for accurate and precise localisation of the tumor and surrounding structures [7]. In patient setup, CBCT is used to confirm the position and alignment of the patient on the treatment couch before each treatment session. This is important because small variations in patient position can lead to significant deviations in the dose distribution delivered to the tumor and surrounding healthy tissue [30]. Image registration algorithms aid in aligning and matching the planning CT images to the cone-beam CT images acquired on the day of treatment ensuring precise and accurate targeting of the tumor volume. Some of the commonly used methods include mutual information-based, normalized cross-correlation-based, and gradient correlation-based algorithms [31]. Deep learning has emerged as a promising technique for image registration in radiotherapy, as it can improve the accuracy and speed of the process [32–34]. It is also widely used to eliminate the artifacts observed on CBCT images [22, 35, 36]. Deep learning architectures such as U-Net, and variations of Generative Adversarial Networks (GANs) have their own strengths and limitations. A study by Fonesca et al. [37] has shown that the use of deep learning to reconstruct and assess the accuracy of HU numbers on the CT image dataset in the region of extended field of view. Our study focusses on first eliminating the streaking artifacts and reconstructing the missing anatomy of CBCT images. The workflow presented in this study uses the pre-treatment CBCT image dataset acquired during the first fraction of radiotherapy treatment. Before the patient returns for their second fraction, a transfer of learning could be performed on the base machine learning model to create their patient-specific model. This model can then be used to improve the quality of the CBCT images taken as the patient is set up for their second fraction and used to improve the accuracy of the patient’s setup. After each subsequent fraction, the patient’s CBCT image dataset grows, and via further transfer learning, an increasingly more accurate model can be produced. Transfer learning could take place routinely between each fraction or until the law of diminishing returns makes it no longer worth the computation time.

To reduce computation time when training a patient-specific model, the performance of the base machine learning model becomes paramount and therefore the number of images, their quality, and overall consistency of the training data are increasingly important. To further reduce variability in the training data, separate models for male and female is recommended. Training data could be split by gender, height, weight, etc. to ensure that the training data is as consistent as possible. When a patient presents for a treatment and a CBCT dataset is acquired, the transfer learning could be performed on the most appropriate base model for that individual to improve the accuracy of the patient-specific model and minimise on-site computation time. With a higher accuracy base model, the computational power required on-site to perform patient-specific transfer learning could be satisfied by current, mid to high-range desktop PCs making it a quick, simple, and routine task performed cost-effectively in a radiotherapy clinic.

The machine learning model used in this research was the pix2pix implementation of a CGAN which is a general-purpose, image-to-image translation model [28]. While this model shows promising utility in the imputation and translation of medical images, it is recommended that other machine learning algorithms be explored or perhaps a bespoke algorithm be developed to achieve faster learning and greater performance. Pre-processing of the data in this research was limited to the CBCT input images only, however, the target images could also be slightly modified to increase the accuracy of the model. In the planning CT datasets used, clothing is visible around the patient. This information is unnecessary for the purpose of the model and is essentially noise in the target image. Removing this noise will increase the quality of the dataset and naturally result in increased performance of the model.

The workflow proposed in this study is suitable for tumors located in a more lateral position and provides a solution to resolve the limitation of missing partial anatomy during CBCT imaging due to a limited field of view [38]. The method is applicable for treating tumors in the breast, lung, and liver, as well as for larger patients.

## Conclusion

It has been demonstrated that modern, general-purpose, and open-source machine learning algorithms have the capability to generate missing anatomy and remove streaking artifacts without the need for highly-expensive purpose-built computers. At present, patient setup using on-couch CBCT imaging is the industry standard and every radiotherapy department using this methodology is doing so using partial CBCT images. The patient setup methodology proposed in this paper has exciting utility in all said radiotherapy departments.
